# The “empty void” is a crowded space: health service provision at the margins of fragile and conflict affected states

**DOI:** 10.1186/1752-1505-8-20

**Published:** 2014-10-22

**Authors:** Peter S Hill, Enrico Pavignani, Markus Michael, Maurizio Murru, Mark E Beesley

**Affiliations:** 1 School of Population Health, The University of Queensland, Herston Road, Herston 4006, Brisbane, Australia; 2 School of Population Health, The University of Queensland, Rua Aquino de Bragança 140, Bairro COOP, Maputo, Mozambique; 3 Alameda Santos 2491/72, 01419-002 São Paulo, Brazil; 4 Via Gagini 4, 41125 Modena, Italy; 5 10 Veronica House, Wickham Road, Brockley, London SE4 1NQ, UK

## Abstract

**Background:**

Definitions of fragile states focus on state willingness and capacity to ensure security and provide essential services, including health. Conventional analyses and subsequent policies that focus on state-delivered essential services miss many developments in severely disrupted healthcare arenas. The research seeks to gain insights about the large sections of the health field left to evolve spontaneously by the absent or diminished state.

**Methods:**

The study examined six diverse case studies: Afghanistan, Central African Republic, Democratic Republic of the Congo, Haïti, Palestine, and Somalia. A comprehensive documentary analysis was complemented by site visits in 2011–2012 and interviews with key informants.

**Results:**

Despite differing histories, countries shared chronic disruption of health services, with limited state service provision, and low community expectations of quality of care. The space left by compromised or absent state-provided services is filled by multiple diverse actors. Health is commoditized, health services are heterogeneous and irregular, with public goods such as immunization and preventive services lagging behind curative ones. Health workers with disparate skills, and atypical health facilities proliferate. Health care absorbs large private expenditures, sustained by households, remittances, charitable and solidarity funding, and constitutes a substantial portion of the country economy. Pharmaceutical markets thrive. Trans-border healthcare provision is prominent in most studied settings, conferring regional and sometimes true globalized characteristics to these arenas.

**Conclusions:**

We identify three distortions in the way the global development community has considered health service provision. The first distortion is the assumption that beyond the reach of state- and donor-sponsored services is a “void”, waiting to be filled. Our analysis suggests that the opposite is the case. The second distortion relates to the inadequacy of the usual binary categories structuring conventional health system analyses, when applied to these contexts. The third distortion reflects the failure of the global development community to recognise—or engage—the emergent networks of health providers. To effectively harness the service provision currently available in this crowded space, development actors need to adapt their current approaches, engage non-state providers, and support local capacity and governance, particularly grassroots social institutions with a public-good orientation.

## Background

The past decade has seen global development actors paying increasing attention to fragile or failed states, reflecting a tension between the demonstrable need, the imperatives for donor investment, and the difficulties in achieving change [1,2]. The 2011 World Bank review heightened concern that “no low-income fragile or conflict-afflicted country has yet to achieve a single United Nations’ Millennium Development Goal” [3]—though limited and inconsistent progress is evident in the recent 2013 analysis [4]. In response, a “New Deal for engagement in Fragile States” was promoted at the Fourth High Level Forum on Aid Effectiveness at Busan [5], with a renewed focus on the contribution of health care to stabilisation and state-building [6], and on meeting immediate health service needs, while simultaneously building governance and capacity in the public healthcare system [7,8].

State fragility has been variously defined—compounding definitions of poor governance, authoritarian rule, transition from war to peace, lawlessness and poverty [9]. Most definitions share an emphasis on state willingness and capacity to assure security and provide essential services, and its effectiveness in achieving this [6]. The prevailing discourse connotes this vulnerability as a transient deviation from the norm, to be corrected by international interventions and, even better, prevented [10]. Yet the history of many fragile and conflict affected countries suggests that the full roles of the state may never have been achieved [11], and that their recurrent and protracted patterns of dysfunction are not transient, and liable to correction, but chronically entrenched.

International development assistance largely proceeds from an implicit assumption of at least basic country-wide health systems managed by legitimate national health authorities, with donors offering political, military or financial support where necessary, and a trajectory that will lead in time to effective state provision of services. Our experience suggests that the focus on the state and its vulnerability conceals a reality that starkly diverges from such an assumption. Healthcare provision is becoming increasingly pluralistic, unplanned, privatized, unregulated and globalised all over the world.

Much of the policy analysis currently available focuses on state-building and the need to develop the state’s capacity to provide essential health services within a viable policy framework. This research shifts that focus, exploring the ways in which healthcare provision is configured by multiple actors beyond the reach of a state absent, unwilling or unable to provide public services. The research draws key lessons learned from six case-studies undertaken between 2010 and 2012 in healthcare arenas under protracted, severe stress.

While specific findings from individual case studies cannot be generalized, the privatisation, commoditisation, and trans-border provision of healthcare are global phenomena, at play also in many peripheral, poor and under-governed situations [12]. The health sector issues in fragile and conflict affected states may not be unique, however, the challenges to comprehensive health service delivery are extreme, and the emergent responses may be more evident, as is the failure of the global development community to effectively address them. With the residual burden of the MDGs about to be carried into the post-2015 Sustainable Development Goals [4], addressing the persisting challenges in least developed countries requires a realistic appraisal of these distressed and complex environments. This paper seeks to contribute to that overview through a synthesis of the evidence from the comprehensive accounts of the six individual case-studies and of the research synthesis report [13], and in other detailed publications [14-16].

## Methods

The research was undertaken by independent investigators with extensive field experience of low-income country contexts, and examined six case-studies of chronically disrupted health sectors, selected for maximum diversity: Afghanistan, Central African Republic, Democratic Republic of the Congo, Haïti, Palestine, and Somalia. The research was funded by the Danish Ministry for Foreign Affairs, and coordinated by the University of Queensland, with ethics approval granted by their Research Ethics Committee (Approval No. 2010001054). The donor had no influence on design, methods or findings of the studies.

Each case study was informed by comprehensive documentary analysis of the available peer-reviewed and “grey” literature—government, bilateral and multilateral agency reports, unpublished research, project reports and evaluations. Particular attention was given to the broader historical, geographical, political, economic and social context in which health care is provided, in the belief the latter is heavily influenced by the former. Field visits in 2011–2012, in all case-studies (with the exception of Somalia) enabled direct observation of the current context, both in the capital and in accessible peripheries. The findings of these case studies were confirmed by subsequent visits to Afghanistan, Haïti and Somalia in 2013–2014. Interviews were undertaken with key informants, with the findings corroborated between members of the research team, and public health experts. Identification of interview participants in each case study was purposive, using government and donor listings, personal networks and subsequent snowballing to access officials, managers, professionals, academics and development practitioners, triangulating evidence from a broad spectrum of sources within the healthcare arena.

The data retrieval and interviews were undertaken using a common thematic guide, developed collaboratively by the researchers. Specific themes were explored in further detail in the country setting where they assumed particular relevance. Over 40 interviews were undertaken in each study. Preliminary findings were analysed by the research team and detailed case-study reports compiled with review by all researchers. Further triangulation of individual case-studies was enabled through the international circulation of draft reports for internal peer review by public health practitioners experienced in each of these locations, and the presentation of the research main findings at a series of international gatherings.

## Results

Recognising the constraints of space, the analysis provided in this paper is necessarily an overview, presenting selected findings from the research reports [12]. The findings are presented in two parts: the first has selected findings from both documentary analysis and site visits that seek to outline the diversity of the case-studies; the second examines the commonalities that were identified in terms of health patterns and outcomes.

### Unique histories, shared outcomes

Given their selection for maximum diversity within the cluster of fragile and conflict affected states, the country case-studies can be expected to demonstrate unique histories of disruption and socio-economic disadvantage. Despite this, they share significant commonalities in terms of governance and service provision in health. The diversity is evident even in their basic descriptive profiles; the commonalities became more apparent on analysis of the findings of the research.

#### Afghanistan

Afghanistan has a centuries-old history of challenges to state-making, attempted by both indigenous and foreign actors. Since 2002, following the displacement of the Taliban by a United States led alliance, major resources have been invested in the building of a liberal state [17]. Within the health sector, the delivery of a basic package of health services (BPHS) with the aim of universal access, has been achieved by competitive contracting-out to non-governmental organisations (NGOs). Access to basic health services increased dramatically from a very low baseline, thanks to the huge expansion of the external resources allocated to health [18]. Reforms in financial management, hospital care, human resource development, and the pharmaceutical sub-sector followed after some delay, and registered less progress. But behind the reassuring screen of the progress registered in the coverage of basic services, the unregulated privatisation of healthcare provision has proceeded [19]. The initial advantage of the relatively well-performing public healthcare system is gradually being lost, owing to reduced access to under-funded public services perceived of inferior quality. All this amidst enduring conflict and insecurity, state deterioration and territorial fragmentation [16,20].

#### Central African republic

The Central African Republic emerged from the “Scramble for Africa” as a colonial cul-de-sac, deprived of strategic or economic value [21]. Gaining independence, it has been characterized as a ghost state [22], unable to broadcast power to its periphery, ensure law and order, build institutions and provide services. Colonial under-investment, followed by internal misrule and compounded by external intervention, have generated widespread social upheaval, stalled development and progressive impoverishment, with ethnic, religious and social divisions reflected in the recent civil conflict. The limited presence of the state has been constant through a succession of regimes [23]. Country-wide, the quality of healthcare provision appears strikingly diverse, with the inevitable concentration in the capital Bangui, and some regions served by international agencies, others by faith-based organisations (FBOs), and some not at all. Severe lack of transport, long distances, impassable or insecure roads and financial barriers further hinder healthcare provision. Traditional healers constitute the most accessible segment of the healthcare market. Drug-selling outlets are commonplace. The bulk of domestic health financing is private, with pervasive unofficial charges even where services are delivered from state-owned premises.

#### Democratic Republic of Congo

The Democratic Republic of Congo (DR Congo), the largest state in sub-Saharan Africa, was carved within awkward colonial borders, which resulted in a constellation of populated peripheries with no core, spontaneously fissiparous, and ungovernable as a unitary state [24]. The long, porous Congolese borders compound the problems of effective governance. From its colonial past, the DR Congo has faced a violent history, marked by state deterioration, extensive privatization of the public realm, and widespread challenges to law and order. Despite this disruption, the state administration survives, yet “[t]he state is so present, but so useless” [25]. Rather than being transitory, this situation seems bound to persist. Policies and plans issued by central health authorities with donor support have a modest impact on actual health service delivery; decentralisation has stalled, failing to deliver on its promises, with scarce resources failing to adequately reach the periphery. As a result, an enormous variation characterises local healthcare provision arrangements, in response to local needs, conditions and actors on the ground [26]. Together, FBOs and NGOs deliver a large portion of health services, according to a variety of procedures, and account for most innovative policy responses. Under-resourced ‘public’ health services adopt “coping” mechanisms that jeopardise coverage and quality. Systemic inefficiencies and financial barriers result in very low outputs. Pervasive commoditisation and medicalization are the natural result of such pressures.

#### Haïti

In Haïti, natural disasters and disease outbreaks, compounded by social divisions, state disarray and political instability, have resulted in a succession of “routinized ruptures” [27]. Born out of a celebrated revolution that established chronic international debt, and deep social inequity between rural and urban communities, Haïtians have maintained a historical ambivalence with respect to their state [28]. The earthquake that struck Haïti in 2010 and the subsequent cholera outbreak have exposed to an extreme extent most of the perennial drawbacks affecting the country: the vulnerability of national institutions, the structural inability of domestic as well as foreign actors to interact productively, the indifference of incoming agencies to the context, the vulnerability of national structures to shocks, and the country’s extreme dependency on external resources. The healthcare arena reflects the broader context: NGOs, FBOs and informal providers deliver most health care in a fragmented and unregulated way, with the Ministry of Public Health and Population relegated to a marginal role. Traditional healing practices offer a widespread alternative. Substantial external resources, used in such a fragmented way, attain modest results.

#### Palestine

Palestine is a nation constrained in its progress to UN recognized statehood, and increasingly partitioned into detached regions maintained in contact by a shared identity, rather than by functioning political and economic links. The multiple barriers conditioning the daily life of Palestinians also define its healthcare arena, which is transnational and pluralistic, and comprising multiple health service delivery systems, spanning the West Bank and Gaza, Israel, Lebanon, Syria and Jordan. Despite recurrent funding crises, there has been a proliferation of healthcare facilities, with this redundant and duplicative situation justified by the parcelling of the territories imposed by Israel, and the constraints on travel between them. In the context of multiple, overlapping policy, planning and regulatory environments, health services of variable—often inadequate--quality have grown in the absence of a coordinated developmental direction, and are sometimes in competition with each other. The aggregated disarray starkly contrasts with the existence of technical, political and social capacity, available across so many organisations and regularly tapped during the frequent crises. The lack of an integrated health system, which only a sovereign state could build, results in multiple health-seeking behaviours across existing borders, according to opportunity, cost, need and appeal.

#### Somalia

Since the implosion of the former Republic of Somalia in 1991, several autonomous regions have emerged, with Somaliland having attained de-facto statehood. The concern with the regional impact of political instability has caused recurrent foreign engagements, with regularly disappointing results. Despite the prevailing turmoil, Somalia functions as an “economy without state” [29], with its vigorous and unregulated merchant activity servicing the region. The healthcare arena is composite, cosmopolitan, and globalised. It has surprisingly managed to grow in spite of multiple constraints, thanks to entrepreneurship, remittances, charitable support and international assistance. Many Somalis travel within and outside Somalia, eclectically choosing from a variety of health services on offer. In such an active environment, crowded with healthcare providers, resource levels, service uptake, quality of care, safety nets, and support systems are vastly better than expected. Grassroots local innovations blend traditional care with modern, Western practice. Travel and internet connectivity provide access to a range of international health services in a highly commercialised and inequitable healthcare market.

### Shared characteristics, unique manifestations

#### Uncertain, unstable polities

Despite their differing histories, the six countries share key characteristics. The disarray in their public health systems is enduring and entrenched. Despite episodic progress, they have been largely resistant to external peace-making and state-building efforts. At the point of writing, Eastern DR Congo remains violent, the CAR is shaken by another civil conflict, while Haïti remains embroiled in the intractable problems of post-quake reconstruction. The future of the state built in Afghanistan by an outside intervention is uncertain as the United States of America withdraws troops. The diversification of Somalia into autonomous regions proceeds, amidst recurrent famines in violence-affected areas. Finally, the Palestinians remain mired in endless conflict, negated human rights, expropriation and poverty.

#### Unreliable services, disrupted access

In each case study, informants reported basic mistrust of state administrations by their citizens. Their inability to provide health and other services may interface with violent and predatory practices. In Haïti, Farmer outlines the links between political violence and public health [30]. Unpaid salaries are recouped directly from patients in the CAR, and the pragmatics of cost recovery in DR Congo leads to a reversal of the direction of duty of care as health authorities seek to cover their costs from patient fees, with “rent” sought in turn from higher levels within the administration [25]. Public services remain concentrated in the capital and other urban centres. Access to publicly-provided, formal health services is limited, with the exception of Palestine, where multiple service options supported by generous aid flows are available. Proper referral pathways may be disrupted or blocked by political and military barriers (as in Palestine), by geographical, financial, and transportation obstacles (as in the CAR), by financial incentives distorting clinical decisions (as in the DR Congo), by violence, sectarian or ethnic mistrust.

#### Health seeking, care provision across borders

The failure of domestic health systems leads to trans-border healthcare provision in response to demand. With porous territorial borders, massive movements of people, ideas, goods, fighters, weapons, health workers, aid agencies, resources, medicines and diseases follow. Migrants and asylum seekers from these countries facilitate a range of trans-border healthcare encounters, by financing movements, advising prospective patients, carrying medicines, returning to the place of origin as providers. Somalia and Palestine offer outstanding examples of trans-border healthcare provision; Afghanistan, the CAR, the DR Congo and Haïti present different brands of the same phenomenon. Given the fluidity of health care provision and health seeking across national borders, the conventional analysis of ‘national’ health systems fails to capture the full extent of complex regional interplays.

#### Unreliable evidence, inequitable responses

The official representations of health services in each of the case studies were inaccurate, and did not expose the deeper and often poorly documented internal diversity within the health system, with national aggregate data concealing disparities in both health provision and health status between regions. In areas affected by violence, well-resourced humanitarian interventions often provided services superior to other regions that were peaceful, but shared an even worse health status. Crises spill over borders, bringing the relief enterprise with them: the CAR received limited development assistance until the Darfur conflict stimulated a new interest in its remote, violent and destitute northern region. Chronic healthcare deficits, exacerbated by acute crises, may be addressed by short-term humanitarian interventions, but these do not necessarily result in long-term health systems development.

#### Limited coordination, inadequate service integration

**Figure 1:**
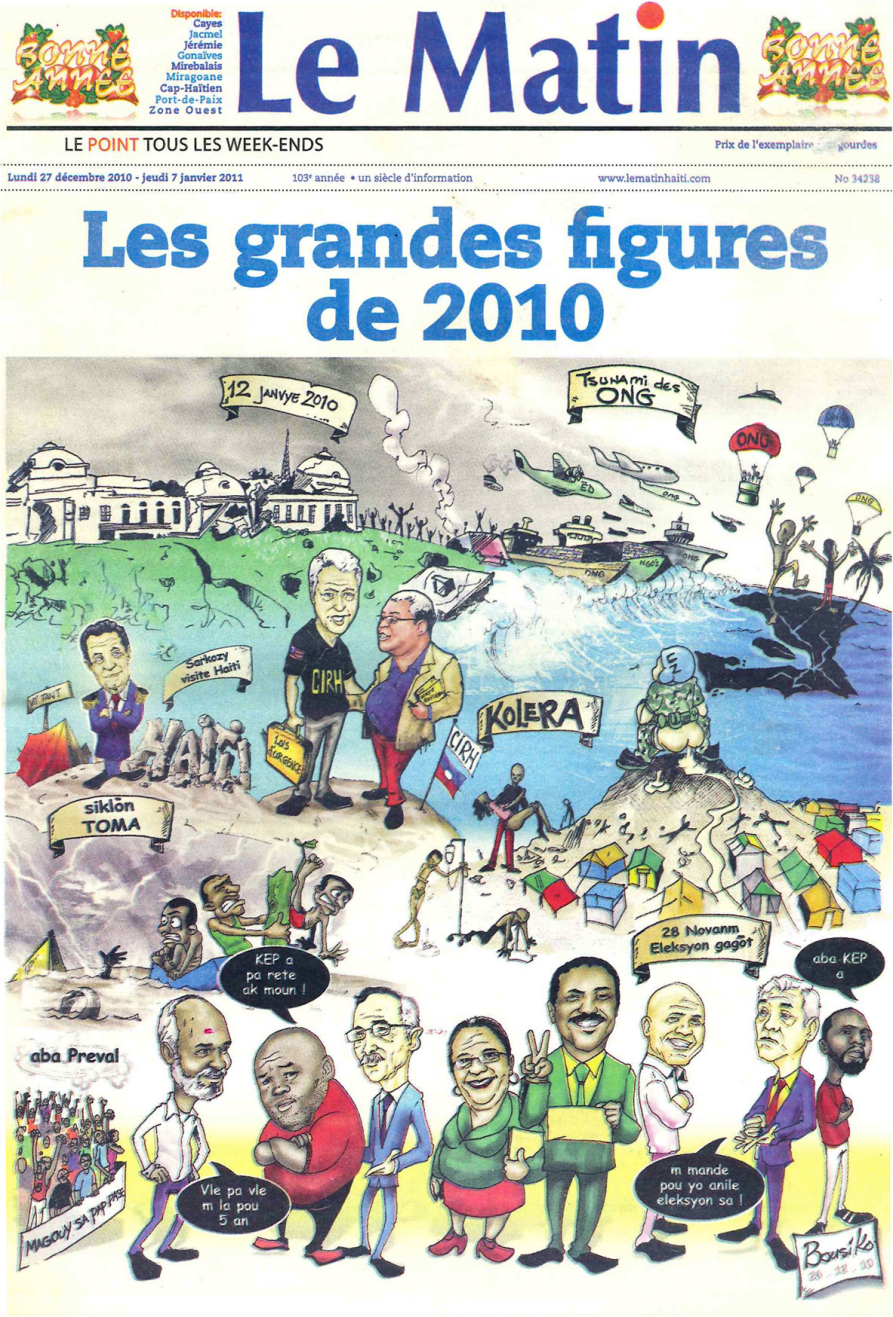
2011 New Year edition of Haïti’s “Le Matin”.

Limited coordination and regulation of non-state healthcare networks was evident in all case studies. In Haïti, the 2010 earthquake resulted in a generous international response, with the Ministry of Public Health and Population—its key staff relocated into a tent city—rapidly overwhelmed by the demands of coordination. The 2011 New Year edition of Haïti’s “Le Matin” captures the local perspective of the cascade of challenges of the previous year: the massive physical destruction compounded by a “tsunami of NGOs”, high profile international political interventions and a persisting cholera epidemic linked to an unpopular UN peacekeeping mission (Figure 1).

Even in more routine circumstances, the non-state segment of the healthcare market is characterised by its diversity, caused by assorted investments by charities, NGOs, donors, local entrepreneurs, politicians and the diaspora. The limited disposable income available means that small, minimally equipped facilities tend to dominate the landscape, with duplication and redundant services leading to unproductive competition. Even where state health plans define the standards or formats for health facilities, those emerging in the non-state sub-sector often fail to conform. Privately-owned facilities not subjected to regulatory criteria tend to evolve organically, adding revenue-fetching equipment and services whenever the opportunity arises. Under-utilisation of state facilities as a result of user fees, poor quality of care, absent staff, unavailable medicines, limited opening time, a lack of transportation and insecurity, make non-state providers competitive, particularly where they show themselves to be more consistently responsive.

#### Emergent healthcare networks, both local and international

The opportunistic growth of private actors to occupy the space left by the receding or absent state produces complex emergent linkages for health services both locally and internationally. Informal but pervasive extended family-based networks ensure the pooled financing of privately-provided health care in unlikely contexts. Small-scale philanthropy is evident in local clinics supported by successful emigrants—at times providing specialist services in isolation from any referral chain. Specific communities may have exceptional health services patched together by rotations of visiting international professionals or through “twinned” institutional support from linked religious organisations. In both Somalia and Palestine, Islamic charities play significant roles, engaging communities over longer time frames, and collaborating with private practitioners by incorporating strategies to protect the poor. Christian FBOs provide a significant component of both primary and secondary care in Haïti, the DR Congo and the CAR. Such fragmentation makes coordination difficult, but at local levels grassroots governance structures may emerge. In situations where NGOs are of sufficient scale and capacity, structured arrangements can take root. Over time, hospitals in Mogadishu have managed to agree to a division of technical competences, which rationalises referrals in the violence-ridden city. East Jerusalem is served by a structured hospital network supported by Muslim and Christian organisations, which avoids competition by specialising facilities, cuts costs and raises quality of care.

#### Imbalanced workforce development training and deployment

The over-production of under-skilled health professionals, who in turn induce an over-supply of poor-quality care, is commonplace. Unregulated training institutions, largely financed by student fees, offer courses demanded by applicants, rather than those needed by health services. The resulting workforces are very imbalanced, with the most common pattern producing a surplus of medical doctors and a shortage of nurses and midwives. Contrary to expectations, state collapse in Somalia has triggered a burgeoning of private health training institutions created in response to market demands, though no schools of pharmacy. Civil services have historically employed large numbers of health workers, providing jobs to new entrants into the labour market, regardless of health service needs, and absorbing most of the scarce public funding for health, but without correcting the essential maldistribution of the workforce. Official payrolls inflated by ghost workers give a very misleading picture of actual staffing patterns in public health facilities. In Katanga (DR Congo) about half the health workers were unregistered volunteers without formal employment, paid directly from user fees. Not all practicing health workers hold recognised qualifications, and many holders of formal qualifications migrate to foreign countries with Haïti an extreme case exporting an estimated 80% of its freshly graduated doctors.

#### Poorly regulated pharmaceutical markets

The pharmaceutical market thrives to take full advantage of the regulatory vacuum, as attested by the proliferation of import–export dealers and selling outlets visible in most settings. Where estimates of total expenses on pharmaceuticals are available, they represent the dominant share of total health expenditure. These markets have regional implications, becoming conduits through which medicines transit unchecked and unrecorded to supply neighbouring countries—undermining their attempts at regulated import, and quality control. Thus, Afghanistan is the natural link between producers in India and Pakistan, and consumers in Central Asia. In turn, pharmaceutical supplies for the Horn of Africa are channelled through Somalia. The recurrent claims that medicines available on the market are counterfeited, repackaged, expired, and dumped are plausible, even if not always backed by solid data. The circulation of sub-standard medicines is a prominent public health concern [31]. Fragmented procurement and supply channels prevail everywhere, with predictable effects on prices, availability and quality of medicines. In Haïti, high quality health services and medicines offered without charge by emergency-oriented NGOs compete with local state health services based on cost recovery through user fees, and the ‘dumping’ of residual supplies as they withdrew, disrupted fragile local pharmaceutical supply networks [32]. In the DR Congo, quality drugs are provided at competitive prices by a non-profit supply agency, but public managers turned private entrepreneurs prefer to purchase cheaper drugs of poorer quality from commercial sources, because of their higher profit margins [33]. Additionally, health authorities often have vested interest in the commercial, unregulated deals they are supposed to regulate.

#### Blurred interfaces between western and folk medicines

Folk medicine cohabitates with and complements biomedical health care in each of the case-studies: in the poorest CAR provinces, it represents the main mode of treatment; in Haïti, traditional medicine was “available to all”. Providers and patients do not draw sharp boundaries between indigenous and imported health care, switching between them on the basis of perceived efficacy, or combining them in “hybrid” treatments. The cost of accessing treatment, the possibility to obtain attention on credit, and the reputation of the available options influence the health-seeking behaviour of the customers. In case of forced displacement, lightly-equipped folk healers may still be able to provide care on the move, or in new settlements. Folk healers borrow drugs, techniques and ideas from Western medicine. The scope of this conspicuous constituent of healthcare is poorly addressed by conventional healthcare analyses, which tend to focus on formal health facilities, and often only those under state administration. But surveys point to substantial health expenses incurred by households to access traditional and “hybrid” health care, usually multiples of the expenditure provided by governments and donors.

## Discussion

The analysis and synthesis of these six case-studies suggests that there are three conspicuous distortions in the way the global development community has considered health service provision in fragile and conflict affected states. The first distortion is the assumption that beyond the reach of state sponsored services is a “void”, waiting to be filled. Our analysis suggests that the opposite is the case. In each case study, the bulk of health services is offered within this “void”: multiple, diverse substitutes have emerged where public services have been absent. As Anderson suggests, “the absence of the state does not simply produce chaos. It also reveals the outlines of alternatives to the state itself” [34]. The available peer-reviewed and “grey” literature—despite their predominant focus on formal, public sector health activities—provide ample evidence of extensive and unexpected activity by private for-profit and not-for-profit providers offering a variety of services. Survey data in these fragile and conflict affected states consistently points to the bulk of health expenditure paid out-of-pocket by households to buy privately-provided health care.

The second distortion is evident in the inadequacy of conventional binary categories, when applied to these contexts [35,36]. The public-private divide is not clear on the ground. Where public health workers either moonlight in private outlets or at home, or supplement their salaries with direct financial demands on public patients, the public/private and formal/informal distinctions are readily blurred. In fact, most state facilities in these case studies depend on formal co-payments—often compounded by informal charges [37,38]. Where funding and consumables unpredictably reach—or do not reach—the periphery, public care is virtually privatized. In these contexts, where such “coping mechanisms” become the norm, concepts like corruption, legality, accountability and governance must be submitted to critical scrutiny [39]. Confidently separating qualified from unqualified staff is also problematic where the quality of training is questionable and the certification of qualifications is inadequate. With the problem of non-rational use of pharmaceuticals, and their adoption into traditional practice, lines between Western and traditional medicine blur. And where international NGOs provide significant contributions to primary and secondary health care—at times deputizing for the state—state/civil society differentiation becomes muddied. The large role of the diaspora makes the domestic/foreign pairing of questionable value. This pervasive blurring, together with the broader problems of data quality and information systems within the health sector, calls for critical analyses of the interface between the public and private sectors that allow this complexity to be adequately unpacked.

The third distortion reflects the failure of the global development community to recognise—or engage—these health providers, often unpredictably linked through international networks emerging in these complex environments. Unregulated contexts, such as fragile and conflict affected states, behave as complex adaptive systems, with the commodification of health and porous borders driving opportunistic international pharmaceutical markets, complicated by the attendant problems of absent quality control and counterfeit drugs. But more benign networks produce local solutions—clinics supported or intermittently staffed by a generous diaspora, and religious philanthropy that ranges in scale from international “twinning” initiatives that link individual church communities to extensive service provision. South-south “trade as aid” functions across a similar diversity of scope; health provision is a focus for the development of international commercial investments, and burgeoning facilities in neighbouring countries service these mobile populations.

The problem of emergent private provision is that in the aggregate it is largely inefficient and often ineffective. Its profit-based motivation distorts the range and distribution of services provided and leaves significant proportions of the population without access to any Western health care. While some gaps are filled with redundant and often duplicative services, others are neglected. Market forces, local demand and parochial planning drive new initiatives, unintegrated into either provincial or national strategies. Public health services are neglected. Private medical schools multiply, but midwifery training lags behind; curative care multiplies opportunistically in response to market demand, available donor funding or specialist expertise. Private initiative is obviously no panacea. Yet this is often the only healthcare provision available in the space beyond the reach of the state and of official external assistance. And emergent private, informal services will continue to compete with greater predictability and flexibility than their state equivalents faced with unstable and dubiously qualified staff complements, and unpredictable salaries, resources and medicines.

## Conclusions

The 4th High Level Forum on Aid Effectiveness in Busan redefined the processes and players that feed into development there are many more sources of development: assistance than those captured by the Organization for Economic Cooperation and Development (OECD) Creditor Reporting System. It recognised the complexity of the evolving development landscape [40,41], the diversity of development stakeholders across both public and private sectors, and confronted the reality that aid alone cannot achieve development [5]. Yet despite this recognition, the “New Deal to engage fragile states” is essentially state-centred in its orientation, with commitments to use the Peace-building and State-building Goals, to focus on country-owned transitions out of fragility, and to provide aid and manage resources more effectively [8].

Clearly, even in the context of the progressive redefining of development effectiveness, that active, unregulated, often chaotic space beyond the reach of the state is going to be an uncomfortable locus for both donors and governments to operate in. Yet, to effectively harness the potential that currently is available in that space, and encourage the emergence of local institutions that will curb some of its shortcomings, regulate quality and validate experience and qualifications, it will be necessary to change the ways in which donors engage fragile and conflict affected states.

We will need different analytic approaches to adequately represent these complex, adaptive and diverse health care environments: current sector reviews, with their focus on the state sector alone, inevitably present a distorted picture of the whole. Planners need on the one hand to open up their scope of enquiry, on the other to seek greater local detail. In contexts where populations are mobile, historical state boundaries may obscure critical elements of the total picture: shifting the focus to populations, to a regional analysis, may enable trans-border elements of healthcare demand and provision to be adequately captured. The local diversity that emerges in these spaces beyond state governance means that sector-wide analyses must be assembled from the bottom up, by studying as many distinct local situations as possible. Identifying local variance is critical to mounting an appropriate health system response: national averages and country-wide generalisations hide rather than reveal the variations existing on the ground. The development of this detailed social mapping offers a first point of engagement with local communities and actors.

But any attempt to comprehensively engage the space beyond the reach of the state will require extensive reorientation of development approaches. Extending the timeframe of donor engagement is imperative: the current dysfunction is chronic, and even where positive progress is evident, reversals will be frequent and sustainable transformation will be incremental. With the diversity of actors and complexity of networks and alliances, identifying appropriate partnerships and building relationships of trust will take time. Funding disbursement will be slow: it will require multiple smaller quanta of financial investment in a mostly decentralized manner. Donors will need to move closer to the populations which they are supporting. These labour-intensive processes will require intimate local knowledge. Progress will be predicated on stable relationships and the gradual strengthening of local services, preserving what is already functioning, even if inadequately. The social institutions on which development is dependent have to grow at the pace allowed by the context, the actors active in it, and the values permeating the social fabric. The success of many FBOs rests clearly on the extent to which they are socially embedded.

A voice must be given to non-state actors, usually as active in the field as they are absent in policy discussions. Private providers, pharmaceutical dealers, training institutions have both a direct knowledge of healthcare provision and a large influence on it, which cannot be ignored. Any exploration will need to identify and strengthen existing networks that have assumed responsibilities normally associated with the state [42]. This burgeoning, diffused governance is evident in all the six case-studies: emigrant professionals who maintain professional links through funding, services or training; long term relationships between FBOs—both Christian and Islamic—and the communities they serve; emergence and self-organisation of services in improbable locations; local councils that take responsibility for establishing their own health and education services; international linkages that provide not only remittances, but emergent services such as web-based clinical and psychiatric consultations.

Donor agencies are already playing an expanded role in each of the case studies, not always overtly acknowledged in the rhetoric of state-building. There is an argument—that may seem superficially to be in tension with the “New Deal”—that would see the global development community enter a dialogue with fragile and conflict affected states to complement their state-building with more direct role in those areas where the state is currently unable to provide services [43]. By recognising the opportunities emerging in the field, development actors may become appropriate “brokers”. The kinds of dialogue that we are advocating are commonplace in developed economies: the state extends its function through its managed relationships with non-state actors. Batley and Mcloughlin [44] have outlined the possibilities for direct and indirect engagement of non-state service providers from service delivery to policy level, commencing with low risk negotiated engagement and progressing with time, capacity and economic development to regulation and contracting, governance functions of the effective state. But the matrix allows for positive engagement from where we are now, transforming the “void”, seeking nodes of possibility in this crowded space.

## Competing interests

The authors declare that they have no competing interests.

## Authors’ contributions

EP, MM, MMu and PH conceptualised the study, and developed the shared methodology and research instruments. EP, MM, MMu, PH and MB planned and undertook the cases studies, and prepared country reports. EP, MM and PH undertook the primary analysis that synthesised the findings of the study. PH and EP prepared the manuscript of this paper. MM, MMu and MB provided comments on the draft and approve its findings. All authors read and approved the final manuscript.
